# Pharmacokinetics of memantine in rats and mice

**DOI:** 10.1371/currents.RRN1291

**Published:** 2012-02-15

**Authors:** Maria G. Beconi, David Howland, Larry Park, Kathryn Lyons, Joseph Giuliano, Celia Dominguez, Ignacio Munoz-Sanjuan, Robert Pacifici

**Affiliations:** ^*^Director, DMPK, CHDI Management/CHDI Foundation, Princeton, NJ; ^†^Director of In Vivo Biology, CHDI Foundation Inc; ^‡^Director, PreClinical Research, CHDI Foundation, Inc; ^§^Pharmacokinetic consultant for the CHDI Foundation, Holland NY; ^¶^CHDI Foundation, Inc.; ^#^VP, Chemistry CHDI Foundation/ CHDI Management Inc. Los Angeles CA; ^**^VP Translational Biology, CHDI Management/CHDI Foundation Inc., Los Angeles (USA) and ^††^CSO - Drug Discovery & Development, CHDI Foundation / CHDI Management

## Abstract

To evaluate the potential of memantine as a therapeutic agent for Huntington’s disease (HD) we have undertaken a series of in vitro, ex vivo and whole animal studies to characterize its pharmacokinetics (PK) and pharmacodynamics (PD) in rats and mice. Results from these studies will enable determination of memantine exposures needed to engage the related functional PD marker and help predict the dose regimen for clinical trials to test its proposed mechanism of action; the selective blockade of extrasynaptic, but not synaptic, NMDA receptors. The studies reported here describe the PK of memantine in rats and mice at low (1 mg/kg) and high (10 mg/kg) doses. Our studies indicate that the clearance mechanisms of memantine in rats and mice are different from those in human, and that clearance needs to be taken into account when extrapolating to the human. In rats only, there is a significant metabolic contribution to memantine clearance at lower dose levels. While memantine is primarily cleared renally in all three species, the proportion of total systemic clearance above the glomerular filtration rate (GFR) is much higher in rats and mice (~13, 4.5, and 1.4 times higher than GFR in rats, mice, and humans, respectively), suggesting that the contribution of active transport to memantine elimination in rats and mice is more significant than in the human. In rats and mice, memantine had a short half-life (<4 h) and steep Cmax/Cmin ratios (>100). In the human, the half-life of memantine was reported to be very long (60-80 h) with a Cmax/Cmin ratio at steady state concentrations of ~1.5. A small change in the clearance of memantine - for example due to renal impairment or competition for the elimination pathway with a co-administered drug - will likely affect exposure and, therefore, the selectivity of memantine on NMDA receptors . The PK differences observed between these species demonstrate that the PK in mice and rats cannot be directly extrapolated to the human. Further, the relationship between the plasma concentration (and therefore dose) needed to elicit a mechanism-related in vivo functional effect (PD readout) while maintaining the selectivity of the extrasynaptic blockade of the NMDA receptors needs to be established before clinical trials can be appropriately planned.

Corresponding author: **Maria G. Beconi, Ph.D**.  Director, Drug Metabolism and Pharmacokinetics CHDI Management/CHDI Foundation. 300 Alexander Park, Suite 110. Princeton, NJ 08540. Tel. 609.945.9055. maria.beconi@chdifoundation.org

## INTRODUCTION

Memantine represents a new class of therapeutic agent with efficacy for the symptomatic treatment of moderately severe to severe Alzheimer’s disease (AD) and which has regulatory approval in the European Union, Australia, and the US. In humans, memantine (10 mg twice daily; C_max _= 83.5 ng/mL or 0.47 µM, in plasma and serum, respectively) was efficacious in a 6-month study treating moderate to severe AD [1], and also demonstrated efficacy in dementia patients (AD patients with Hachinski Ischemia Scale scores ≤4) at a 10 mg single daily dose for 12 weeks [2]. In a rat AD model, memantine improved cognition [1] and elicited neuroprotective effects [1] [3]. However, the plasma or brain concentrations associated with these effects were not defined in these studies.

There is interest in the scientific community in evaluating memantine’s potential as a therapeutic agent in the treatment of Huntington’s disease (HD) via preferential blockade of extrasynaptic NMDA receptors (NMDARs). Synaptic and extrasynaptic NMDARs induce different signaling cascades, activating cell-survival and -death pathways, respectively [4]. Evidence suggests that NR2B-containing NMDARs contribute to the degeneration of medium spiny neurons (MSNs) in the striatum of HD patients [5] [6], and NR2B-containing extrasynaptic NMDARs have been shown to have increased surface expression, current, and toxicity in striatal MSNs in HD mouse models [5] [7] [8]. Increased NMDAR signaling observed in YAC128 mice occurs before motor dysfunction and neuronal loss, and is correlated with a shift in the subcellular distribution of  the NR1, NR2a, and NR2b subunits of the NMDAR complex from synaptic to extrasynaptic membranes [5]. This shift in the balance towards increased extrasynaptic activity appears to drive neuronal dysfunction by impairing the CREB-PGC1a cascade, which results in reduced nuclear CREB phosphorylation, and by increasing levels of Rhes protein [9], a small guanine nucleotide binding protein recently shown to be involved in sumoylation and disaggregation of mutant huntingtin (HTT) [10] [11].  

Memantine is an NMDA antagonist that at low dosage preferentially blocks extrasynaptic but not synaptic NMDAR signaling [9]. Low-dose memantine (1 mg/kg in drinking water) for 2 months reversed the deficit in CREB phosphorylation, with a concomitant improvement in a rotarod learning task in YAC128 mice [5]. These effects were seen at a very early age (4 months) in the YAC128 mouse after 2 months of low-dose memantine exposure.

Low-dose memantine was also shown to rescue neuropathology as well as motor deficits in aged (12 months) YAC128 mice [9]. Interestingly, high-dose memantine (30 mg/kg) worsened motor performance and neuropathology compared to vehicle-treated mice; these detrimental effects seem to be consistent with memantine at this dose blocking NMDAR activity in the synapse [9]. Specifically, NMDAR activation at the synapse elicited changes consistent with neuroprotection, including induction of mutant HTT inclusions though a TRiC chaperonin-dependent mechanism that relies on TCP-1 expression. While low-dose memantine preserved the TRiC dependent pathway, high-dose memantine was found to decrease HTT aggregate formation and TCP-1 expression levels [9]. In addition, low- (but not high-) dose memantine resulted in decreased striatal levels of Rhes protein, which would be predicted to have beneficial effects by regulating mutant HTT aggregation and clearance.  

Taken together, these studies indicate that memantine provides protection in an HD animal model, although a narrow window of efficacy versus toxicity is evident. In these mouse studies, memantine exposures that elicit the observed effects were not determined and the mechanism by which memantine elicited the response was not correlated to drug concentrations; in the absence of this information it is not possible to determine target human exposures. The differential function of synaptic and extrasynaptic NMDARs (induction of cell-survival or -death pathways, respectively) and the fine balance of memantine action suggested by its narrow efficacy window means that dosing regimens will be a critical factor in designing a clinical trial with the best possibility of testing memantine efficacy via its proposed mechanism in HD patients. It is therefore imperative that the correlation between the PD markers of memantine action and associated exposures in preclinical models are precisely defined to enable extrapolation to the human.

To increase the probability of success in a clinical trial of memantine, a defined dose-dependent PD marker is required to correlate dose/effect between preclinical models and the human. Studies described above suggest that TCP-1 and pCREB are directly affected by preferential extrasynaptic blockade of NMDARs and should be evaluated as potential PD markers. Our research strategy for memantine includes:

1. Characterization of the metabolism and PK of memantine in rats and mice, and correlation to the human
2. Development of *ex-vivo* assays to define a PK/PD response in animal models
3. Establishment of dose-dependent PD endpoints in animal models directly related to the mechanism of extrasynaptic blockade, which can also be used in clinical trials.

In this report we characterize the metabolism and PK of memantine in rats and mice and correlate that to the human. Results from these studies will be used to guide further modeling of the PK and PD profiles of memantine in rodents and extrapolate that to human exposure.   

## MATERIALS AND METHODS

### Pharmacokinetic Studies

The goals of these studies were to evaluate the PK of memantine (hydrochloride salt) in Sprague-Dawley rats and C57Bl/6 mice following a single intravenous bolus (iv; 1 mg eq./kg), oral gavage (po; 1 and 10 mg eq./kg), or subcutaneous (sc; 1 and 10 mg eq./kg) administration. 

To assist with the interpretation of the rodent and human PK parameters, the following *in vitro* ADME studies were conducted: (a) stability of memantine in rat, mouse, and human liver microsomes (b) metabolite identification in rat liver microsomes (c) permeability in Caco2 cells (d) direct and indirect transporter uptake studies to determine whether memantine is a substrate for human uptake transporters.

####     Compound synthesis

Memantine (also denoted CHDI-00051501-0001-004 in this report) was synthesized using published procedures.

####     Animals and housing

 The animal phase of these studies was conducted at Xenometrics. Xenometrics is committed to the highest standards of laboratory animal welfare and is subject to legislation under the Animal Welfare Act.  Xenometrics is fully accredited by the Association for Assessment and Accreditation of Laboratory Animal Care International (AAALAC), and is registered with the United States Department of Agriculture (USDA).  All procedures involving animals were conducted humanely and were performed by or under the direction of trained and experienced personnel.  The protocol was reviewed and approved by the IACUC of Xenometrics prior to study initiation.  The veterinarian was consulted in the overall study design for this study type.  The study performed under this protocol did not unnecessarily duplicate previous studies.  There were no significant changes to the protocol involving animals, however, should there have been, they would have been implemented after IACUC approval. These studies were conducted under protocol No 65730-3, CHDI A‑3535, dated 31-Mar-2010.

Sprague Dawley (SD) rats (138 adult males), and C57Bl/6 mice (140 adult males), were obtained (Hilltop Laboratories, Scottsdale, PA); 123 per species were used in the study and the remainder provided blank matrix. Animals were individually identified by tail markings and were acclimated to the study environment for 16-21 days prior to dose administration. Animals were individually housed in suspended wire caging, and were kept on a 12h/12h light/dark cycle except when interrupted for study procedures. Average room temperature was regulated in the range 18 to 29°C, average relative humidity of 30-70%, and an average daily airflow >10 fresh air changes/h. Animals were fed LabDiet^Ò^ Certified Rodent Diet 5002 Meal food ad libitum, except during fasting prior to dose administration, and had access to water ad libitum. 

####     Test article and formulation preparation

Memantine was dosed as a solution in physiological saline as a single dose via intravenous bolus (1 mg eq./kg), oral gavage (po; 1 and 10 mg eq./kg), or subcutaneous (sc; 1 and 10 mg eq./kg) administration. The concentration of memantine in the 1 mg eq./kg iv, po, and sc solutions dosed to rats was 1 mg eq./mL, and the dose volume was 1 mL/kg. The concentration of memantine for the same dose level administered to mice was 0.2 mg eq./mL, and the dose volume was 5 mL/kg. The concentration of memantine in the 10mg eq./kg po and sc solutions was 5 mg eq./mL and the dose volume was 2 mL/kg for both rats and mice.

####     Dose administration and sample collection

Details of the dose administration, body weights, clinical observations, plasma and brain sample collection and preparation are not shown in this report but are available upon request. Blood (for preparation of plasma) and brain samples were collected at 0.08, 0.25, 0.5, 1, 2, 4, 8, and 24h post-dose. 

####     Pharmacokinetic analysis

Since all time points were terminal, mean memantine concentrations per time point were used to calculate the composite PK parameters by non-compartmental analysis using WinNonlin program, version 5.2 (Pharsight Corp., Mountain View, California). A model was selected based on the vascular (iv bolus) or extravascular (po or sc) routes of administration. For the iv route, plasma concentration at time zero was back extrapolated from the first two observed post dose plasma concentrations. For the po and sc routes, concentration at time zero was assumed to be zero. Plasma and tissue concentrations below the limit of quantitation were treated as absent samples for the purpose of calculating the mean plasma concentration values or for calculating PK parameters.

The area under the plasma concentration versus time curve (AUC) was calculated using the linear trapezoidal method (linear interpolation). When appropriate, the terminal elimination phase of the PK profile was estimated based on the best fit (r^2^) using at least the last three observed concentrations.  PK parameters describing the systemic exposure of memantine in plasma and brain were estimated from observed (rather than predicted) plasma/brain concentrations, the dosing regimen, the AUC, and the terminal elimination phase rate constant (k_el_) for each group. The portion of the AUC from the last measurable concentration to infinity was estimated from the equation Ct/k_el_, where Ct represents the last measurable concentration. The extrapolated portion of the AUC was used for the determination of AUC_0-∞_. The percent bioavailability (%F) was calculated by dividing the dose normalized extravascular plasma AUC_0-∞ _by the dose normalized iv plasma AUC_0-∞_ times 100. The bioavailability calculations assumed concentrations were in the linear range.

Throughout this report, first pass metabolism refers to the amount of drug that is metabolized or transported out in the intestine and liver during the absorption process and therefore does not reach systemic circulation.

####     Bioanalysis

Concentrations of memantine in rat and mouse plasma and brain were determined using an LC-MS/MS assay developed at ABC Laboratories. Calibration standards were prepared by adding memantine to control plasma or control brain homogenate obtained from Sprague-Dawley rats or C57Bl/6 mice. Three calibration curves were prepared using concentrations from 1.00-5000 ng eq./mL for plasma, 0.500–2500 ng eq./mL for brain (1 and 1.22 mg eq/kg doses) and 1.00-5000 ng eq./mL for brain (10 mg eq/kg doses).  The lower limit of quantitation (LLOQ) corresponded to the lowest acceptable calibration standard for each assay and were 5.58 nM for rat and mouse plasma, 9.76 nM for rat brain, and 19.5 nM for mouse brain. Econazole nitrate was used as an internal standard in the LC-MS/MS assay. Complete details on assay methodology can be obtained from the authors. The structures of the analyte (memantine) and the internal standard are shown in **Figure 1**.

**Figure:**
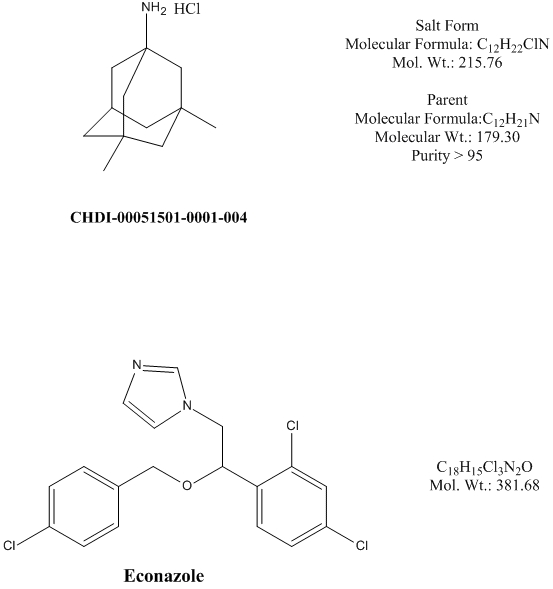
**Figure 1.  Structures of memantine (CHDI-00051501-0001-004) and internal standard (econazole nitrate) used in this study for LC-MS/MS analysis.** 

### In Vitro Metabolism Studies

####     Incubations in hepatic microsomes

Memantine (1 μM) was incubated in 0.5 mg/mL of pooled male rat, mouse, or human liver microsomes (obtained commercially), in 0.1 M phosphate buffer at 37ºC. After pre-warming the mixture for 5 min, reactions were initiated by the addition of NADPH (1 mM). Aliquots (80 μL) were taken at 0, 5, 10, 20 and 40 min, and the reaction immediately terminated by adding into 80 μL of acetonitrile containing an analytical internal standard (IS). Samples were centrifuged and the supernatant fractions analyzed by LC-MS/MS with multiple reaction monitoring (MRM). All incubations were performed in duplicate. Testosterone was used as the positive control. The MRM area response ratio of the analyte over IS for Time=0(control) was set to 100%, the relative decrease in MRM area ratio intensity over time against that of the control (percent parent decrease) was used to determine the half-life (t_1/2_) of memantine in the incubation. Half-life values were calculated from the relationship: 

t_1/2_ (min) = -0.693/λ, where λ is the slope of the Ln concentration vs time curve. The intrinsic clearance (Cl_int_) was calculated as: 

Cl_int_ = (0.693 x incubation volume (μL))/(t_½_ (min) x mg of microsomal protein)

####     Metabolite identification

Metabolites of memantine were characterized following incubation in pooled rat liver microsomes. Incubation conditions were as described above (Section 4.1), except that for metabolite identification the memantine concentration was 10 µM, 1 mg/mL of liver microsomal protein was used, and the metabolites were identified following a 60 min incubation period. Control incubations (T=0 min) were used as reference, and testosterone incubations were used as a positive control.

Full scan mass spectra were acquired by TOF-MS as accurate mass. The instruments were controlled and data acquired by Waters MassLynx™ version 4.1 (SCN 714) software.  The TOF-MS spectra were interrogated and metabolite reports generated by Waters MetaboLynx™ XS software.

### Permeability and Transporter Studies

####     Bidirectional Caco permeability

CacoReady ^TM^ 24-transwell plates with plated Caco cells were purchased from Advancell, and were prepared for the experiments following manufacturer’s instructions. Memantine and reference compounds (propranolol, digoxin and vinblatine) were added to either the apical or basolateral chambers of the transwell plate assembly at a concentration of 10 μM prepared in Hanks’ Balanced Salt Solution containing 25 mM HEPES (pH7.4). Lucifer Yellow (LY) was added to the donor buffer in all wells to assess viability of the cell layer. Since LY cannot freely permeate lipophilic barriers, results were rejected when a high degree of LY transport (>100nm/s) was observed. After a 1 h incubation at 37°C, aliquots were taken from both chambers and added to acetonitrile containing an analytical internal standard in a 96-well plate. Concentrations of compound in the samples were measured by (HPLC-MS/MS). 

P_app_ values were calculated from the relationship: 

        P_app_ = ([compound]_acceptor final_ × V_a_ )/ (A × time ([compound]_donor initial_ )

Where,

        V_a_ = Volume in acceptor compartment

        A = Surface area o n the cell membrane

The efflux ratio (Er) was calculated as

        Er = P_app_ (B to A) / P_app_ (A to B)

The following assay acceptance criteria were used:

        Propranolol: P_app_ (A>B) value ≥ 20(×10^-6^cm/s)

        Vinblastine: P_app_ (A>B) value < 5 (×10^-6^cm/s) with Efflux ratio ≥5.

        Lucifer yellow permeability: ≤100nm/s

####     Indirect uptake transporter assay: 

####  To determine whether memantine is an inhibitor of the human OATP2B1 (OATP-B), OCT1, OCT2, OAT3, PEPT1, PEPT2 uptake transporters. The standard uptake transporter assay is an indirect, inhibitory-type screening assay, performed with cold test article, which in this case was memantine. It provides information on any interaction (substrate or inhibitor) between selected uptake transporters and memantine that would affect the transport of the reporter compound. This assay does not give information on the nature of the interaction (transported substrate or inhibitor). A radiolabeled reporter substrate is transported into the cells transfected with the selected human uptake transporter, and the inhibitory effect of memantine on the transport of this reporter substrate is measured. Values are presented on a relative scale; 100% defined as transport in the presence of the solvent and without memantine (no inhibition), and 0% defined as transport detected without transporter activity. IC_50_ is defined as the concentration required to inhibit the transport of the reporter substrate by 50%.

The uptake transporter assay was carried out under the conditions described in the table below, with either CHO cells or MDCKII cells stably expressing the respective uptake transporter.   

**Treatment groups in the indirect uptake transport assays**

**Table:** 

**Transporter**	**Incubation time** (min)	**Probe substrate **(concentration)	**Reference inhibitor** (concentration)
human OATP2B1	15	E3S (1 μM)	Fluvastatin (10 μM)
human OAT3	5	E3S (0.3 µM)	Probenecid (500 µM)
human OCT1	10	TEA (3.6 μM)	Verapamil (100 μM)
human OCT2	10	Metformin (2 µM)	Verapamil (100 µM)
human PEPT1	10	Gly-Sar (1.8 μM)	Tyr-Phe (200 μM)
human PEPT2	10	Gly-Sar (1.8 μM)	Cefadroxil (200 μM)

*E3S: estrone-3-sulfate, TEA: tetraethylammonium chloride, Gly-Sar: Glycylsarcosine, Tyr-Phe:Tyrosine-Phenylalanine. The viability of the transporters was evaluated using the respective reference substrate and inhibitor of the transporter.*

Duplicate incubations of memantine (0 or DMSO control, 0.41, 1.2, 3.7, 11, 33, 100 and 300 µM) were conducted per concentration on transfected and in parental cells. A reference inhibitor (positive control, duplicate) was incubated in transfected cells.

CHO cells were cultured in a 1:1 mixture of Dubelcco’s Modified Eagle’s Medium (DMEM, Lonza, Allendale, NJ) and Ham's F-12 (F-12, Lonza, Allendale, NJ) and MDCKII cells in DMEM at 37°C in an atmosphere of 95:5 air:CO_2_. Cells were plated onto standard 96- or 24-well tissue culture plates in DMEM F-12 medium supplemented with 5 mM sodium butyrate for CHO cells or DMEM supplemented with 10 mM sodium butyrate for MDCK cells and were incubated for 24 h (there is no butyrate induction in the case of OAT3, OCT1, OCT2, PEPT1 and PEPT2).

Before initiating the experiments, the medium was removed, cells were rinsed with 2 x 100 µl of freshly prepared Krebs-Henseleit buffer (Sigma-Aldrich, St Louis, MO). Uptake experiments were carried out at 37^o^C in 50 µl of Krebs-Henseleit buffer (pH 5 in case of PEPT1 and PEPT2) containing the probe substrate and memantine or the solvent. The same organic solvent concentration, which did not exceed 1% (v/v), was used in all wells.

After the experiment cells were rinsed with 2 x 100µl of Krebs-Henseleit buffer and lysed with 50 µl of 0.1M NaOH. ^3^H-estrone-3-sulfate, ^14^C-TEA, ^14^C-metformin and ^3^H-Gly-Sar transport was determined by total radioactivity by transferring an aliquot (35µl) from each well to a vial containing liquid scintillant for analysis.

OAT3 cells were plated onto standard 24-well plate in a density of 2 x 10^5^ cells/well. The washing steps were performed with 2 x 200 µl of Krebs-Henseleit buffer. OAT3 cells were treated with 200 µl 5 mM glutaric acid before the initiation of the experiment. Uptake was evaluated at 37^o^C in 200 µl of Krebs-Henseleit buffer containing the probe substrate and memantine or the solvent. The final concentrations of memantine were 0 (or DMSO control), 11, 33, 100 and 300 µM. For the cell lysis 200 µl NaOH (0.1 M) were used and 150 µl aliquot were transferred to vials containing liquid scintillant for analysis. Parental cell lines served as negative controls. 

####     Direct uptake transporter assay: 

To determine whether memantine is a substrate of the human OATP2B1 (OATP-B), OCT1, OCT2, OAT3, PEPT1, PEPT2 uptake transporters.

The potential for memantine to be a substrate of an uptake transporter was determined directly by comparing the uptake of memantine into cells expressing the selected uptake transporter with its uptake into control cells. Memantine was added to transporter-expressing and control cells at two concentrations (PEPT1 and PEPT2 100 and 300 µM, OCT1 and OCT2 50 and 100 µM, OATP2B1 100 and 300 µM), and incubated with cells for 2 and 20 min at 37±1°C (75 µL final incubation volume). The final concentrations of memantine were selected to be the IC_50_ of reporter substrate transport inhibition established in the indirect transporter assay and one order of magnitude lower. Incubations were carried out as described in the indirect uptake transporter assays. The concentration of memantine was determined by LC-MS/MS. 

## PHARMACOKINETIC STUDY RESULTS

### Pharmacokinetics in rats

A summary of the PK parameters obtained following iv, po, or sc dose of memantine to rats is shown in **Table 1**. The plots of the mean memantine plasma and brain concentrations over time are shown in **Figure 2** and **Figure 3**, respectively. Dose formulation analysis indicated that the actual concentration of memantine was within ±15% of the nominal concentration of 1 mg/mL (sc) and 5 mg/mL (po and sc). The 1 mg/mL iv and po dose formulation, however, was 122% of nominal, thus, for these dose levels, the dose was adjusted to 1.22 mg eq./kg for the PK calculations. 

**Table 1.  Summary of pharmacokinetic parameters following an iv, oral, or subcutaneous dose of memantine to rats.**

Doses (solutions in saline) = iv: 1.22 mg eq./mL; po: 1.22 and 10 mg eq./mL; sc: 1 and 10 mg eq./mL.

**Table:** 

**PK PARAMETERS**	**UNITS**	**RAT PLASMA**				
		**IV** ** ** **1.22 mg eq./kg**	**PO** ** ** **1.22 mg eq./kg**	**PO** ** ** **10.0 mg eq./kg**	**SC** ** ** **1.0 mg eq./kg**	**SC** ** ** **10.0 mg eq./kg**
AUC_(0__-__last)_	nM hr/kg	1573	540.8	11250	1616	25780
AUC_(0-__¥__)_	nM hr/kg	1638	594.0	11390	1686	25810
AUC_norm_	(nM hr/kg) /(kg/mg)	1342	486.9	1139	1686	2581
Bioavailability (F)	%	NA	36.3	84.9	125.6	192.3
Observed C_max_	nM	NA	108.7	3696	985.0	6189
Observed C_max_Norm	nM/ (mg/kg)	NA	89.07	369.6	985.0	618.9
Observed T_max_	hr	NA	1.0	0.5	0.25	0.5
Plasma Clearance (CL_p_)	L/hr/kg	4.15	NA	NA	NA	NA
Mean Residency Time (MRT_0-∞_)	hr	2.08	NA	NA	NA	NA
Volume of Distribution at Steady State (Vd_ss_)	L/kg	8.62	NA	NA	NA	NA
Half-life (t_½_)	hr	1.90	2.23	3.92	1.81	2.59
Half-life Regression Time Points	hr	2, 4, 8	2, 4, 8	4, 8, 24	1, 2, 4, 8	2, 4, 8, 24
**PK PARAMETERS**	**UNITS**	**RAT BRAIN**				
		**IV** ** ** **1.22 mg eq./kg**	**PO** ** ** **1.22 mg eq./kg**	**PO** ** ** **10.0 mg eq./kg**	**SC** ** ** **1.0 mg eq./kg**	**SC** ** ** **10.0 mg eq./kg**
AUC_(0__-__last)_	nM hr/kg	43000	13060	253500	47970	440900
AUC_(0-__¥__)_	nM hr/kg	47630	21120	256200	52980	441600
AUC_norm_	(nM hr/kg) /(kg/mg)	39040	17310	25620	52980	44160
Observed C_max_	nM	10980	2165	25430	10620	75440
Observed C_max_Norm	nM/ (mg/kg)	9003	1775	2543	10620	7544
Observed T_max_	hr	0.5	2.0	2.0	0.5	1.0
Half-life (t_½_)	hr	2.28	<2.54*	3.70	2.51	2.65
Half-life Regression Time Points	hr	4, 8, 24	2, 4, 8	4, 8, 24	1, 2, 4, 8	4, 8, 24
						
Ratio of Brain-to-Plasma AUC_(0-__¥__)_		29.08	35.56	22.49	31.42	17.11

NA: PK parameters not applicable for this dose route. 

* Value was estimated using the LOQ for the 24 hr concentration; thus, the value carries large imprecision.

**Figure:**
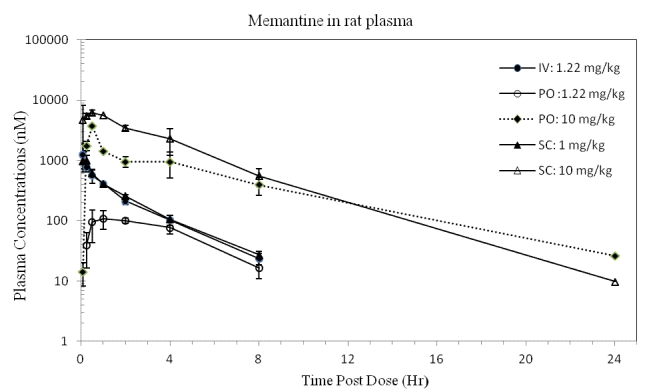
**Figure 2.  Concentrations of memantine (CHDI-00051501-0001-004) in rat plasma over time. (iv 1.22 mg eq./kg, po 1.22 and 10.0 mg eq./kg, sc 1.00 and 10.0 mg eq./kg) (Values represent mean and standard deviation; N=3 per time point)**

**Figure:**
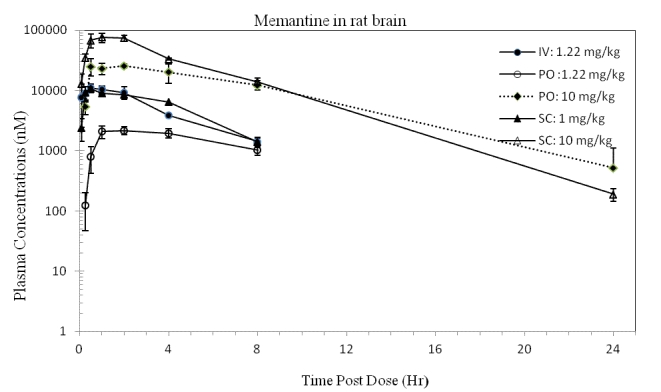
**Figure 3. Concentrations of memantine (CHDI-00051501-0001-004) in rat brain over time. (iv 1.22 mg eq./kg, po 1.22 and 10.0 mg eq./kg, sc 1.00 and 10.0 mg eq./kg) (Values represent mean and standard deviation; N=3 per time point)**

####     IV pharmacokinetics

Following iv administration to rats, plasma clearance was high (4.15 L/hr/kg), the volume of distribution at steady state was large (8.62 L/kg) and the elimination half-life was 1.9 h. The high estimates of plasma clearance could be a consequence of the high volumes of distribution observed, and may not reflect the actual systemic clearance.

####     Extravascular pharmacokinetics

Following extravascular administration, absorption was rapid (T_max_ observed between 0.5 and 1 h). Exposures increased in a more dose proportional manner with dose level, suggesting saturation of first pass (po only) and clearance mechanisms.

With a po dose increase from 1.22 to 10 mg/kg, the C_max_ (nM) increased from 108.7 to 3696 (~4-fold more than dose proportional) and AUC_0-∞ _(nM hr/kg) from 594 to 11390 (2.3-fold more than dose proportional). Following sc administration, C_max_ (nM) increased from 985 to 6189 (slightly less than dose proportional) and AUC_0-∞ _(nM hr/kg) from 1686 to 25810 (1.5-fold more than dose proportional) with a dose increase from 1 to 10 mg/kg. The less than dose proportional increase in C_max_ was attributed to a slower time-to-absorption reflected by the protracted high concentrations at the earlier time points for the 10 mg/kg sc dose. The AUC_0-24_ and AUC_0-∞ _were similar (extrapolation was minimal), suggesting AUC_0-24_ were complete and represented the maximal exposure achieved with that dose regimen. The more pronounced effect of dose level on dose proportionality for the po dose vs the sc dose is attributed to the contribution of first pass in the po dose.

The plasma elimination half-life (t_1/2_) after extravascular administration increased from 2.23 to 3.92 h (po) and from 1.81 to 2.59 h (sc) when doses increased from 1 to 10 mg/kg. This increase is possibly driven by saturation of clearance mechanisms at the highest dose levels, and consistent with the more than dose proportional exposures. The very rapid absorption suggests that contributions from drug absorbed to the plasma concentration observed in the latest time points were negligible, thus the extravascular t_1/2_ is a good estimate of the elimination t_1/2_.

When dose levels increased from 1 to 10mg/kg, the extravascular bioavailiability value (%F) increased from 36.3 to 84.9% after the oral doses, and from 125.6 to 192.3% for the sc doses. Calculation of an extravascular bioavailability assumes concentrations are in the linear range and clearance mechanisms have not changed. Since clearance mechanisms appear to be saturated at the highest concentrations for this drug, the calculation of bioavailability is not valid; values are still reported for illustration purposes. For the oral dose, the increased bioavailability value reflects a combination of saturation of first pass metabolism plus saturation of the clearance mechanisms, with a very pronounced first pass at the lowest dose. 

####     Concentrations in brain

Memantine distributed preferentially to brain tissue, with brain-to-plasma ratios increasing from approximately 3 to >20 over time for all doses and routes (data not shown). The increase in brain-to-plasma ratios over time was driven by a delayed absorption of compound to brain (~0.25 - 1.5 h delay in T_max_ for brain when compared to plasma at the same dose level and route) and not by a longer elimination half-life.

In the brain, the more than dose-proportional increase in C_max_ and exposures were not as pronounced as those observed in plasma, suggesting that distribution of compound to the brain may be close to reaching saturation at the higher doses. With a po dose increase from 1.22 to 10 mg/kg, the C_max_ (nM) increased from 2165 to 25430 (~1.5-fold more than dose proportional) and AUC_0-∞ _(nM hr/kg) from 21120 to 256200 (1.5-fold more than dose proportional). After similar dose increases with sc administration, C_max_ (nM) increased from 10620 to 75440 and AUC_0-∞ _(nM hr/kg) from 52980 to 441600; both increases were slightly less than dose proportional (0.7-0.8-fold).

Consistent with the more than dose-proportional increases in exposures in plasma being more pronounced than in brain, the ratios of brain-to-plasma AUC_0-∞ _decreased when the dose level increased from 1 to 10 mg eq./kg, from 35.56 to 22.49 for the po dose and from 31.42 to 17.11 for the sc dose.

### Pharmacokinetics in mice

A summary of the PK parameters obtained following an iv, oral, or sc dose of memantine to mice is shown in **Table 2**. The plots of the mean memantine plasma and brain concentrations over time are shown in **Figure** **4** and **Figure** **5**, respectively. Dose formulation analysis indicated that the actual concentration of memantine was within ±18% of the target concentration for all dose levels; therefore the target dose levels were used for the PK calculations. 

**Table 2.  Summary of pharmacokinetic parameters following an iv, oral, or subcutaneous dose of memantine to mice.**

Doses (solutions in saline) =  iv: 0.2 mg eq./mL; po: 0.2 and 5 mg eq./mL; sc: 0.2 and 5 mg eq./mL

**Table:** 

**PK PARAMETERS**	**UNITS**	**MOUSE PLASMA**				
		**IV** ** ** **1.0 mg eq./kg**	**PO** ** ** **1.0 mg eq./kg**	**PO** ** ** **10.0 mg eq./kg**	**SC** ** ** **1.0 mg eq./kg**	**SC** ** ** **10.0 mg eq./kg**
AUC_(0__-__last)_	nM hr/kg	1392	1174	18230	1384	21900
AUC_(0-__¥__)_	nM hr/kg	1465	1239	18270	1433	21950
AUC_norm_	(nM hr/kg) /(kg/mg)	1465	1239	1827	1433	2195
Bioavailability (F)	%	NA	84.6	125	97.8	150
Observed C_max_	nM	NA	477.0	7599	526.9	7983
Observed C_max_Norm	nM/ (mg/kg)	NA	477.0	759.9	526.9	798.3
Observed T_max_	hr	NA	0.5	0.25	0.25	0.25
Plasma Clearance (CL_p_)	L/hr/kg	3.81	NA	NA	NA	NA
Mean Residency Time (MRT_0-∞_)	hr	2.35	NA	NA	NA	NA
Volume of Distribution at Steady State (Vd_ss_)	L/kg	8.94	NA	NA	NA	NA
Half-life (t_½_)	hr	1.97	1.86	2.92	1.64	3.07
Half-life Regression Time Points	hr	2, 4, 84, 8	1, 2, 4, 8	2, 4, 8, 24	1, 2, 4, 8	4, 8, 24

**Table:** 

**PK PARAMETERS**	**UNITS**		**MOUSE BRAIN**					
			**IV** ** ** **1.0 mg eq./kg**	**PO** ** ** **1.0 mg eq./kg**	**PO** ** ** **10.0 mg eq./kg**		**SC** ** ** **1.0 mg eq./kg**	**SC** ** ** **10.0 mg eq./kg**
AUC_(0__-__last)_	nM hr/kg		36460	29660	311200		23600	351800
AUC_(0-__¥__)_	nM hr/kg		36590	29770	312300		25770	352800
AUC_norm_	(nM hr/kg) /(kg/mg)		36590	29770	31230		25770	35280
Observed C_max_	nM		7698	4783	67680		5360	72520
Observed C_max_Norm	nM/ (mg/kg)		7698	4783	6768		5360	7252
Observed T_max_	hr		1.0	1.0	1.0		1.0	1.0
Half-life (t_½_)	hr		2.98	3.02	3.05		2.16	3.00
Half-life Regression Time Points	hr		2, 4, 8, 24	4, 8, 24	4, 8, 24		2, 4, 8	4, 8, 24
						
Ratio of Brain-to-Plasma AUC_(0-__¥__)_		24.98		24.03		17.09	17.98	16.07

NA: PK parameters not applicable for this dose route. 

**Figure:**
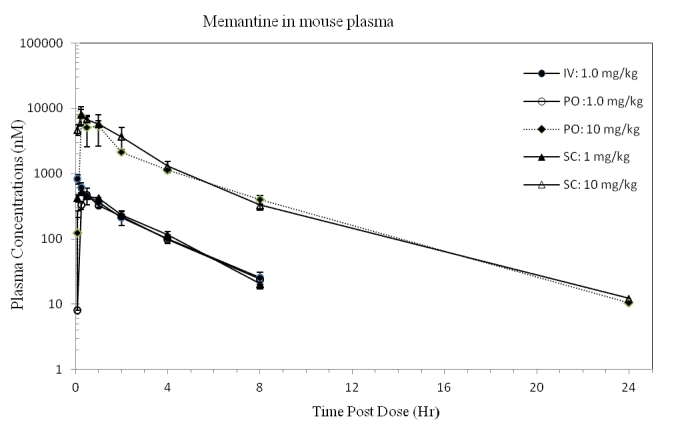
**Figure 4.  Concentrations of memantine (CHDI-00051501-0001-004) in mouse plasma over time. (iv 1.00 mg eq./kg, po 1.00 and 10.0 mg eq./kg, sc 1.00 and 10.0 mg eq./kg) (Values represent mean and standard deviation; N=3 per time point).**

**Figure:**
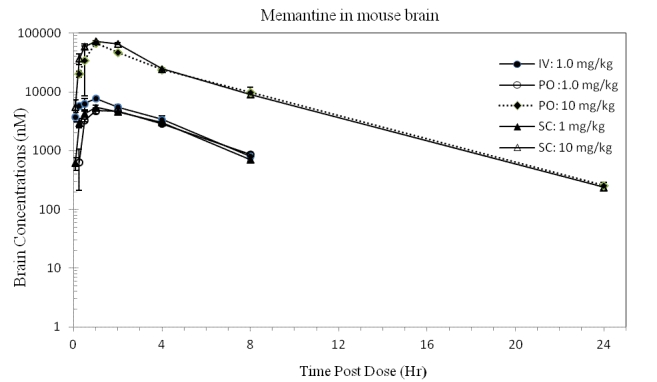
**Figure 5. Concentrations of memantine (CHDI-00051501-0001-004) in mouse brain over time. (iv 1.00 mg eq./kg, po 1.00 and 10.0 mg eq./kg, sc 1.00 and 10.0 mg eq./kg) (Values represent mean and standard deviation; N=3 per time point).**

####     IV pharmacokinetics

Following iv administration to mice, plasma clearance was high (3.81 L/hr/kg), the volume of distribution at steady state was large (8.94 L/kg) and the elimination half-life was 1.97 h. As in rats, the high estimate of plasma clearance could be a consequence of the large volume of distribution observed, and may not reflect the actual systemic clearance.

####     Extravascular pharmacokinetics

Following extravascular administration, absorption was rapid (T_max_ observed between 0.25 to 0.5 h). There was a ~1.5-fold more than dose-proportional increase in both C_max_ and exposures, suggesting primarily saturation of clearance mechanisms. The contribution of saturation of first pass to the more than dose proportional increase in exposures is likely minimal, since the oral bioavailability was high (%F = 84.6% at 1mg/kg po) and exposures were similar for the same dose level between po and sc dose routes. With a po dose increase from 1 to 10 mg/kg, C_max_ (nM) increased from 477 to 7599 and AUC_0-inf_ (nM hr/kg) from 1239 to 18270. For a sc dose increase from 1 to 10 mg/kg, the C_max_ (nM) increased from 526.9 to 7983 and the AUC_0-∞ _(nM hr/kg) from 1433 to 21950. The AUC_0-24_ and AUC_0-∞ _were similar (extrapolation was minimal), suggesting AUC_0-24_ were complete and represented the maximal exposure achieved at this dose level. 

The plasma elimination half-lives after extravascular administration increased from 1.86 to 2.92 h (po) and from 1.64 to 3.07 h (sc) when doses increased from 1 to 10 mg/kg, consistent with the more than dose-proportional exposures and with the hypothesis of saturation of clearance mechanisms with increasing dose levels. Similar to observations in the rat, the very rapid absorption suggests that contributions from drug absorbed to the plasma concentration observed in the latest time points were negligible, thus the extravascular half-life is a good estimate of the elimination half-life.

When dose levels increased from 1 to 10 mg/kg, the extravascular bioavailability (%F) increased from 84.6 to 125% after the oral doses, and from 97.8 to 150% for the sc doses. These values suggest very good-to-complete absorption from both the po and sc doses at 1 mg/kg, with minimal first pass effect for the po dose. As explained for the rat, the oral bioavailability calculation was not done under linear conditions, however, values are presented for illustration purposes. The increase in oral bioavailability to 125% and 150% at 10 mg/kg for the po and sc dose, respectively, is attributed to saturation of the clearance mechanisms. Given the high oral bioavailability of the 1 mg/kg po dose, contributions of saturation of first pass metabolism are likely small. 

####     Concentrations in brain

The distribution of memantine to mouse brain was very similar to that observed in the rat, with brain-to-plasma ratios increased from approximately 1 at earlier time points to >20 at the later time points for all dose levels and routes. The more than dose proportional increase in C_max_ and exposures observed in the plasma were not as evident in the brain, suggesting that distribution of compound to brain may be close to reaching saturation at the higher doses. It is worth noting that exposures achieved at 10 mg/kg in the mouse were within 20% of those previously observed in the rat and similar for both the po and sc routes. With a po dose increase from 1 to 10 mg/kg, the C_max_ (nM) increased from 4783 to 67680 (~1.4-fold more than dose proportional) and AUC_0-∞ _(nM hr/kg) from 29770 to 312300 (dose proportional). After similar dose increases with sc administration, C_max_ (nM) increased from 5360 to 72520 (~1.4-fold more than dose proportional) and AUC_0-∞ _(nM hr/kg) from 25770 to 352800 (~1.4-fold more than dose proportional). 

Consistent with the more than dose proportional increases in exposures in plasma being more pronounced than in brain, the ratios of brain-to-plasma AUC_0-∞ _decreased when the dose level increased from 1 to 10 mg eq./kg from 24.03 to 17.09 for the po dose and from 17.98 to 16.07 for the sc dose.

The brain elimination half-lives after extravascular administrations did not display the same increase with dose level observed for plasma half-lives, and were relatively similar between dose levels and dose routes (~2.16 - 3.05), suggesting that clearance mechanisms from the brain were not saturated and had not changed at the doses administered.  

### Summary of pharmacokinetics in mice and rats

In the rat, the lower oral bioavailability at the lower dose was explained by metabolism during absorption. In both species, memantine has very good oral bioavailability at the highest oral dose.  The greater than 100%F values observed after the sc dose are likely an artifact of the saturation of metabolism during the drug’s elimination phase, observed at both dose levels but which becomes more exacerbated at the highest dose.

The elimination half-life of memantine in rats and mice was short (<4 h); this and the complete exposures at 24 h suggest that, after a single dose, steady state concentrations are achieved within one day. If administered as a single dose at 24 h intervals, the plasma exposure to parent drug will remain relatively constant following multiple doses, provided there is neither induction nor autoinhibition of clearance mechanisms.

Memantine distributed well to brain tissue in both species, with brain-to-plasma ratios increasing from 3 to >20 over time post-dose. The brain elimination half-lives after extravascular administrations did not display the same increase with dose level observed for plasma half-lives, and were relatively similar between dose levels and dose routes (~2.3-3.8), suggesting that clearance mechanisms from brain did not change and were not saturated at increased dose levels.

Similar to plasma, the relatively short half-lives in brain together with complete exposures at 24 h suggest that, after a single dose, steady state concentrations are achieved in brain within one day. Thus, if administered as a single dose at 24 h intervals, the brain exposure to parent drug will remain relatively constant following multiple doses, provided there is no induction or autoinhibition of clearance mechanisms. Since it appears that compound distribution to brain was achieving saturation at the higher doses, doses in excess of 10 mg/kg will reach less than dose-proportional exposures in the brain.

## IN VITRO METABOLISM RESULTS

### Liver microsomal stability

Memantine was relatively stable in human and mouse liver microsomes but underwent extensive oxidative metabolism in the rat. The intrinsic clearances (Cl_int_) calculated from the rate of metabolism of memantine in the liver microsomal incubations were slow for mice and human (<13.9 µL/min/mg protein), but moderate to high for rat (50.4 µL/min/mg protein), suggesting that memantine is a substrate for rat-specific CYPs, which are sex specific for this species(**Table 3**). Incubations were performed using a pool of male liver microsomes. Studies to determine whether the male rat specific CYP2C11 is responsible for the metabolism, or whether metabolism occurs to the same extent in the female rat, are ongoing.

**Table 3. Stability of memantine in mouse, rat, and human liver microsomes**  

**Table:** 

**Species**	**Intrinsic Clearance (Cl**_**int**_ **) **			
	**Liver Microsomes **			
	**(µL/min/mg prot) **			
	**Low**	**Medium**	**High**	**Memantine **
Human	<7	7-40	>40	<13.9
Rat	<13	12-70	>70	50.4
Mouse	<20	20-110	>110	<13.9

#### Identification of metabolites in rat liver microsomes

In rat liver microsomes the most abundant metabolite (based on uv signal response) was the product of a single aliphatic hydroxylation. Additional single aliphatic hydroxylations and *N*-oxide formation were also observed. Given the high degree of symmetry in the molecule and absence of functional groups, the precise location of the metabolic reaction could not be determined from LC-MS/MS analysis (**Figure 6**).

**Figure:**
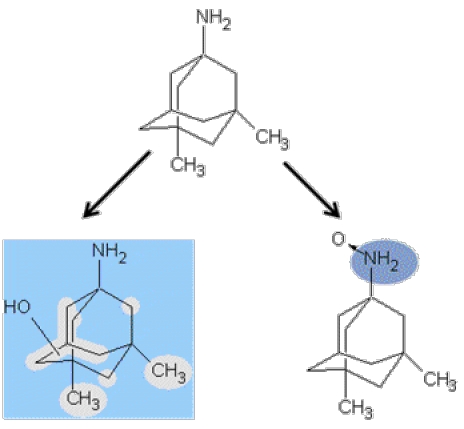
**Figure 6. Identification of metabolites following memantine incubatoins in rat liver microsomes.** 

Memantine metabolism in rat liver microsomes  

### Permeability and transporter assay results

#### Permeability in Caco2 cells

Memantine was highly permeable in Caco2 cells. The AtoB ratio and BtoA ratios were 43.4 and 47.0E^-6 ^cm´sec^-1^, respectively, and was not a substrate for efflux transporters (ER~ 1) (**Table 4**). 

**Table 4. Memantine permeability in Caco2 cells**  

**Table:** 

**Caco Permeability**		**ER**
**E** ^**-6**^ ** (cm x sec** ^**-1**^ **)**		
**AtoB**	**BtoA**	
43.4	47	~1
High perm. >10 E^-6^ cm x sec^-1^		

#### Effect of memantine on the transport of probe substrates for selected uptake transporters

Memantine inhibited the uptake of substrates mediated by OCT transporters (**Table 5**). It was a potent inhibitor of OCT1-mediated TEA (tetraethylammonium chloride) transport with an IC_50_ of 3.5 µM and inhibited the human OCT2-mediated metformin uptake by 97% at the highest investigated concentration (300 mM), with an IC_50_ of 8.9 mM.

**Table 5. Effect of memantine on the uptake of probe substrates for selected uptake transporters.**

**Table:** 

**Test Article**	**Transporter**	**Probe substrate **(concentration)	**IC**_**50**_ ** (µM)**	**Maximal Inhibition (%)**
**Memantine**	PEPT1	E3S (1 μM)	281.5	~62
	PEPT2	E3S (0.3µM)	ND	~22
	OATP2B1	TEA (3.6 μM)	> 300	~67
	OAT3	Metformin (2µM)	ND	NDe
	OCT1	Gly-Sar (1.8 μM)	3.512	~94
	OCT2	Gly-Sar (1.8 μM)	8.862	~97

*ND: Not Determined (< 50% inhibition).* *NDe: Not Decisive (Activation or inhibition > 25% of the baseline but no trend observed).* *NIO: No Interaction Observed (< 20% inhibition).* *Activation: Activation or stimulation of the baseline (%).* *Maximal Inhibition (%): Maximal inhibition observed of the control activity (0% inhibition).* *IC50: test article concentration needed to inhibit maximal activity by 50%* . *E3S: estrone-3-sulfate, TEA: tetraethylammonium chloride, Gly-Sar: Glycylsarcosine, Tyr-Phe:Tyrosine-Phenylalanine. The viability of the transporters was evaluated using the respective reference substrate and inhibitor of the transporter.*

Memantine was also an inhibitor of the peptide transporters; it was a potent inhibitor of the human PEPT1*-*mediated glycylsarcosine transport (IC_50_ = 280 mM) and elicited some mild inhibition (22% at 300 mM) of the PEPT2-mediated glycylsarcosine transport. Memantine did not interact with the human OAT3 mediated estrone-3-sulfate transport, but inhibited the humanOATP2B1*-*mediated estrone-3-sulfate transport by 67% at 300 mM.

#### Evaluation of memantine as a potential substrate for selected uptake transporters

Since memantine interacted with the uptake of probe substrates for PEPT1, PEPT2, OCT1, OCT2, and OATP2B1, follow-up direct uptake experiments were performed to determine if memantine was substrate of these transporters (**Table 6**). No preferential accumulation of memantine was observed in PEPT1- or PEPT2-transfected cells over parental cells, suggesting it is not a substrate of these uptake transporters. In these studies, memantine was not actively transported by OCT1, but appeared to be a substrate of OCT2 and OATP2B1, with an accumulation of memantine in transporter-transfected cells >1.5-fold higher than in the corresponding parental cells.

**Table 6. Evaluation of the potential for memantine to be a substrate of selected uptake transporters.**  

**Table:** 

	**Accumulation in transporter transfected cells (pmol/mg)**				**Accumulation in parental cells (1) (pmol/mg)**				**Highest Fold Activation**
**Transporter**	**2 min**		**20 min**		**2 min**		**20 min**		
	**100 µM**	**300 µM**	**100 µM**	**300 µM**	**100 µM**	**300 µM**	**100 µM**	**300 µM**	
PEPT1	1073	2290	1087	1682	969	2389	981	1614	1.1 (100µM, 20 min)
PEPT2	937	1871	897	1759	965	2011	1219	2056	0.97 (100µM, 20 min)
	**50 µM**	**100 µM**	**50 µM**	**100 µM**	**50 µM**	**100 µM**	**50 µM**	**100 µM**	
OCT1	3818	5878	5540	9190	3894	6337	5672	9576	Not observed
OCT2	8492	15071	8866	15495	5660	8000	5790	8459	1.88 (100µM, 2 min)
	**100 µM**	**300 µM**	**100 µM**	**300 µM**	**100 µM**	**300 µM**	**100 µM**	**300 µM**	
OATP2B1	11317	18296	20820	45866	6409	10891	10889	21496	2.1 (300 µM, 20 min)

(1) Parental cells were CHO cells except for OATP2B1 where MDCKII cells were used as parental cells

## DISCUSSION

Synaptic and extrasynaptic NMDARs are thought to induce different signaling cascades, activating cell-survival and -death pathways, respectively [12]. Previous studies have shown that memantine has a very narrow therapeutic window [9] [5], therefore dose selection will be critical to achieve the exposures that will maximize the probability of selectively blocking the cell-death pathway in a clinical trial. The sensitivity of NMDARs to memantine concentrations has not been clearly established; that is, the range of concentrations *in vitro* that selectively affect the extrasynaptic cascade without affecting the synaptic one is not well defined. Further, the PD response in animal models (that is, the relationship between plasma concentrations and the magnitude of the response of biomarkers that are specific to cell-survival and -death pathways) is unknown. This is significant from both an efficacy standpoint, since it is critical to know that memantine is affecting the pathway of interest *in vivo*, and a patient safety standpoint since in the absence of this information it is not possible to predict what exposure will induce cell-death pathways. As discussed above, increased extrasynaptic activity of NMDARs affects nuclear CREB phosphorylation and TCP-1 expression. Together with collaborators, we are currently developing assays to measure the effects of NMDAR blockade on these two markers *in vitro* and *ex vivo*. The ultimate goal is to correlate the exposures to memantine in animal models to the response in CREB phosphorylation and TCP-1, so that a clear PK/PD correlation can be defined. With this information, we will be better prepared to establish the range of exposures that need to be achieved in humans to selectively affect extrasynaptic response and select the dose regimen accordingly.   

The experiments reported here describe the first step in this process, which is to describe the PK of memantine in rats and mice (species that will be used as animal models) and the difficulties in directly extrapolating these PK parameters to the human. In mice and rats, memantine distributes preferentially to the brain, with brain-to-plasma ratios ranging from around 3 to >20 over time for all dose levels and routes. This increase in brain-to-plasma ratios was driven by a delayed absorption of compound to brain (~0.25 to 1.5 h delay in T_max_ for brain compared to plasma at the same dose level and route) and not by a longer half-life in brain. The plasma exposures and C_max_ after extravascular administration are greater than dose-proportional, and consistent with saturation of clearance mechanisms (both dose routes) and of first pass metabolism (po dose only). 

The greater than dose-proportional increase in C_max_ and exposures observed in the plasma were not so evident in the brain, suggesting that distribution of memantine to brain may be close to reaching saturation at the higher doses. Consistently, the ratio of brain-to-plasma AUC_(0-__¥__)_ decreased when the dose level increased from 1 to 10 mg eq./kg for both the po and sc routes.  The brain elimination half-lives after extravascular administrations did not display the same increase with dose level observed for plasma half-lives, and were relatively similar between dose levels and dose routes (~2.28 – 3.8), suggesting that clearance mechanisms from brain were neither saturated nor had changed at the doses administered.  

In both the brain and plasma compartments, memantine has a relatively short elimination half-life (<4 h for all dose routes and levels) which together with complete exposures observed at 24 h suggest that, after a single dose, steady state concentrations are achieved within one day. Therefore, if administered as a single dose at 24 h intervals the plasma and brain exposure to parent drug will remain relatively constant following multiple doses, provided there is no induction or autoinhibition of clearance mechanisms. Since it appears that compound distribution to brain was achieving saturation at the higher doses, doses in excess of 10 mg/kg will reach less than dose proportional exposures in brain.

The PK characteristics of memantine were similar in rats and mice, except that the compound has a pronounced first pass effect in the rat (36% oral bioavailabiltiy) but not in the mouse (85% oral bioavailability). The more significant first pass effect observed in rats is attributed to oxidative metabolism in this species due to the consistency with the high Cl_int_ observed in liver microsomal incubations. Sex differences in rat CYPs exist; both *in vivo* and *in vitro* studies were carried out in male rats, so it is unclear whether the male rat specific CYP2C11 is responsible for the metabolism observed, or whether metabolism occurs to the same extent in the female rat. Studies to address this question are ongoing. The increase in oral bioavailability in the rat with a dose increase from from 1 to 10 mg eq./kg is explained by saturation of CYP-mediated metabolism, which becomes negligible at the higher dose. 

Consistent with the high absorption observed in rodents and the high distribution to brain tissue, memantine had very high permeability in Caco2 cells and was not a MDR1 (Pgp) substrate. Memantine, however, appeared to be a substrate of the uptake transporters OCT2 and OATP2B1.

While the PK characteristics of memantine in the two rodent species studied here is similar, it is different from that observed in humans. In humans, the efficacious dose of memantine is 10 mg/day BID po (20 mg total dose). Its associated C_max_ in patients with normal renal clearance (Cl_r_) is 83.5 ng/mL plasma/serum (0.47 uM). The compound has complete and rapid absorption (T_max_ ~4-6 h), a large volume of distribution at steady state (Vd_ss_, 9-11 L/kg), and is primarily eliminated via the kidney (75-90% of the dose is eliminated as intact memantine in the urine). Total plasma clearance (Cl_p_) is 182 mL/min, of which 90%  is accounted for by renal clearance (Cl_r_ = 164 mL/min) in individuals with normal renal function. This value of renal clearance is approximately 30% higher than the human glomerular filtration rate (GFR) of 125 mL/min, suggesting active secretion. Severe renal impairment affects memantine elimination, with renal clearance accounting for 92 and ~60-67% of the total systemic clearance under acidic or alkaline conditions, respectively. In the human, the elimination half-life is long (~60-80 h) and it therefore takes a few days of dosing for drug concentrations to reach steady state [13] [14] [15] [16].

A cross-species comparison of the PK (Table 7) indicates clearance needs to be taken into account when extrapolating to the human, since:

- Memantine is cleared primarily via renal elimination (filtration plus secretion) in mice, rats, and humans. However, there is a significant metabolic contribution to memantine clearance in rats at lower dose levels.
- The proportion of total systemic clearance above the GFR is much higher in rodents (~13 and 4.5-fold higher than GFR in rats and mice, respectively) than in humans (~1.4-fold higher than GFR).
- The above data indicates that the contributions of the different clearance mechanisms of memantine to the total systemic clearance is different in rodents vs humans. Contribution of active transport is much lower in humans (30-40%) than rodent (>100%) which probably at least partly accounts for the very different elimination half-life observed (~3-4 h in rats and mice and ~60-80 h in humans).
- Greater than dose-proportional exposures are likely a consequence of changes in clearance mechanisms (saturation of oxidation in rats, and possibly saturation of active elimination in mice and rats).
- In rats and mice, memantine had a short half-life (<4 h), and steep C_max_/C_min_ ratios (>100). In the human, the half-life of memantine was reported to be very long (60-80 h), with a C_max_/C_min_ ratio at steady state concentrations of ~1.5. 

 **Table 7. Pharmacokinetic summary: Cross-species comparison**    

**Table:** 

			**Species**								Rodent: single dose in solution
			**Rat**			**Mouse**			**Human**		
**Dose**	**(mg/kg)**		1	10		1	10		10 mg		Human: BID
											
**AUC**_**inf**_	**(uM hr)**		0.6	11.4		1.2	18.2				More than dose prop exposures
**C**_**max**_	**(uM)**		0.1	3.7		0.5	7.6		0.47		­ in oral t1/2 suggest change in clearance mechanisms
**C**_**min**_	**(uM)**			0.03			0.01		0.3		
**C**_**max/**_ **C**_**min **_ **ratio**				123			760		1.54		Much lower C_max_/C_min_ ratio in human
**T**_**max**_	**(hr)**		1	0.5 - 1		0.5	0.5 - 1		4 - 6		
			**1 mg/kg (iv)**			**1 mg/kg (iv)**					
**Vd**_**ss**_	**(L/kg)**			8 - 9			8 - 9		9 - 11		
**Cl**_**p**_	**(L/hr/kg)**			4.15			3.81		0.16		Much lower (estimated) clearance in human. Renal elimination in all species
**GFR**	**(L/hr/kg)**			**0.31**			**0.84**		**0.11**		Cl_p_ >> GFR suggests active elimination
**t**_**1/2**_	**(hr)**			4			3		60 - 80		

Complete PK parameters can be found in Tables 1 and 2 for rat and mice   

The PK differences observed between these species demonstrate that the PK in mice and rats cannot be directly extrapolated to the human. In addition, the significant contribution of renal clearance (with contributions of active elimination) to the total clearance of memantine in the human suggests that memantine exposures will be altered in patients with renal impairment or under co-medications that compete with memantine for active elimination, possibly resulting in memantine concentrations that are outside the selectivity range of the effect on the NMDARs.

The PD studies now planned will aim to define the plasma concentrations of memantine (and the dose regimen needed to achieve them) that are required to elicit a mechanism-related *in vivo* functional effect (PD readout) in animal models. This will be critical to determining the memantine dose range in humans that will maximize the probability of selectively blocking the extrasynaptic NMDAR cell-death pathway without affecting synaptic cell-survival in any future clinical trial.****

**Acknowledgments**

The authors gratefully acknowledge the contribution and dedication of the scientific staff at the contract research organizations Xenometrics, Solvo Biotechnology, and Evotec. The authors also gratefully acknowledge Simon Noble for the outstanding job in the technical writing of this manuscript.    

## **Competing interests**

CHDI Foundation is a not-for-profit biomedical research organization exclusively dedicated to discovering and developing therapeutics that slow the progression of Huntington’s disease. CHDI Foundation conducts research in a number of different ways; for the purposes of this manuscript, all research was conceptualized, planned, and directed by CHDI scientific staff and conducted at the contract research organizations Xenometrics, Solvo Biotechnology, and Evotec.   

CHDI Foundation provides financial support to *PLoS Currents: Huntington Disease*. Editorial responsibility for the content in *PLoS Currents: Huntington Disease* rests entirely with the Public Library of Science, the Editors, and Board of Reviewers. 
